# Sensitivity of the Cervical Disc Loads, Translations, Intradiscal Pressure, and Muscle Activity Due to Segmental Mass, Disc Stiffness, and Muscle Strength in an Upright Neutral Posture

**DOI:** 10.3389/fbioe.2022.751291

**Published:** 2022-04-27

**Authors:** Rizwan Arshad, Hendrik Schmidt, Marwan El-Rich, Kodjo Moglo

**Affiliations:** ^1^ Biomechanics Laboratory, Department of Mechanical and Aerospace Engineering, Royal Military College of Canada, Kingston, ON, Canada; ^2^ Julius Wolff Institute, Berlin Institute of Health, Charité—Universitätsmedizin Berlin, Berlin, Germany; ^3^ Healthcare Engineering Innovation Center, Department of Mechanical Engineering, Khalifa University, Abu Dhabi, United Arab Emirates

**Keywords:** cervical spine, musculoskeletal model, inverse dynamics, intervertebral disc loads, intradiscal pressure

## Abstract

Musculoskeletal disorders of the cervical spine have increased considerably in recent times. To understand the effects of various biomechanical factors, quantifying the differences in disc loads, motion, and muscle force/activity is necessary. The kinematic, kinetic, or muscle response may vary in a neutral posture due to interindividual differences in segmental mass, cervical disc stiffness, and muscle strength. Therefore, our study aimed to develop an inverse dynamic model of the cervical spine, estimate the differences in disc loads, translations, intradiscal pressure, and muscle force/activity in a neutral posture and compare these results with data available in the literature. A head–neck complex with nine segments (head, C1–T1) was developed with joints having three rotational and three translational degrees of freedom, 517 nonlinear ligament fibers, and 258 muscle fascicles. A sensitivity analysis was performed to calculate the effect of segmental mass (5th to 95th percentile), translational disc stiffness (0.5–1.5), and muscle strength (0.5–1.5) on the cervical disc loads (C2–C3 to C7–T1), disc translations, intradiscal pressure, and muscle force/activity in a neutral posture. In addition, two axial external load conditions (0 and 40 N) were also considered on the head. The estimated intradiscal pressures (0.2–0.56 MPa) at 0 N axial load were comparable to *in vivo* measurements found in the literature, whereas at 40 N, the values were 0.39–0.93 MPa. With increased segmental mass (5th to 95th), the disc loads, translations, and muscle forces/activities increased to 69% at 0 N and 34% at 40 N axial load. With increased disc stiffness (0.5–1.5), the maximum differences in axial (<1%) and shear loads (4%) were trivial; however, the translations were reduced by 67%, whereas the differences in individual muscle group forces/activities varied largely. With increased muscle strength (0.5–1.5), the muscle activity decreased by 200%. For 40 vs. 0 N, the differences in disc loads, translations, and muscle forces/activities were in the range of 52–129%. Significant differences were estimated in disc loads, translations, and muscle force/activity in the normal population, which could help distinguish between normal and pathological cervical spine conditions.

## Introduction

Musculoskeletal disorders such as neck pain are frequent across all age and sex groups. Globally, neck pain prevalent cases were 288.7 million in 2017 (Safiri et al., 2020). Several factors could contribute to neck pain, such as sedentary lifestyle, sustained or awkward posture, vibration, and psychological or socioeconomic factors (Linton, 2000; Charles et al., 2018). Not all but several causes may be linked with a biomechanical condition of the cervical spine (Kong et al., 2017). For example, neck pain may arise from spinal cord compression due to degenerative changes in the spinal structures (Cohen, 2015; McCormick et al., 2020).

To distinguish between asymptomatic (pain-free) population with physiologically intact structures and symptomatic population with a pathomorphological condition, knowledge of the variation in the cervical spine loads, motion, and muscle activity in asymptomatic population is crucial. Previously, experimental studies (Panjabi et al., 1986, 2001; Wheeldon et al., 2006; Ackland et al., 2011; Suderman and Vasavada, 2017) measured load-displacement behavior, muscle moment arm, or the range of motion in flexion, extension, lateral bending, or axial rotation. In addition, computational studies either based on the finite element (Mesfar and Moglo, 2013; Bredbenner et al., 2014; Mustafy et al., 2014; Lasswell et al., 2017), inverse dynamic (Anderst et al., 2013; Diao et al., 2018, 2017), or forward dynamic models (Sartori et al., 2014; Silvestros et al., 2019) investigated the effect of variation in geometrical or material properties on the cervical spine loads, motion, or muscle force. However, in an asymptomatic population, quantification of the differences in spinal loads, segment translations, or muscle activity in a neutral posture needs further attention. Such data provided could help improve the prognosis and outcome of the interventions applied to prevent or treat pathological conditions of the cervical spine.

The anthropometric and biomechanical characteristics in the general population are subject-specific (Vasavada et al., 2008; Winter 2009). Such interindividual variations may lead to significant differences in segmental kinematics, disc loads, or muscle force/activity in a neutral posture. Therefore, we hypothesize that parameters such as segment mass, intervertebral disc stiffness, and muscle strength significantly affect the kinematic, kinetic, or muscle response of the cervical spine in a neutral posture. For example, the cervical segments and head mass vary considerably among the general population. The head has the largest mass, which could vary due to differences in head circumference (Ching, 2007). In addition, the head mass could differ significantly in a specific percentile population of a certain height and body mass (Nguyen et al., 2012). The disc stiffness plays a vital role in flexibility and load-bearing mechanism. Previously, *in vitro* studies showed a large variation in cervical disc axial and shear stiffness (Moroney et al., 1988; Yoganandan et al., 2001; Dowling-Medley et al., 2020). These differences may influence the local kinematics, the initial contact mechanics between the facet joints (Yoganandan et al., 2003; Jaumard et al., 2011), and the cervical spine’s overall motion and load sharing mechanism (Cripton, 1999; Patwardhan et al., 2000). In addition, neck muscle strengths vary widely among the population (Ikai and Fukunaga, 1968; Maganaris et al., 2001; Catenaccio et al., 2017), leading to significant differences in the level of muscle activity required to stabilize the cervical spine in a neutral posture.

In the asymptomatic population, quantification of the differences in the cervical disc loads, disc translations, and neck muscle force/activity due to variation in segmental mass, disc stiffness, and muscle strength still requires more consideration. Therefore, the aim of this study was 1) to develop an inverse dynamic musculoskeletal model of the cervical spine, 2) calculate the differences in cervical disc loads, disc translations, intradiscal pressure, and the muscle force/activity due to segmental mass, translational disc stiffness, and muscle strength in a neutral posture, and 3) compare these results with data available in the literature.

## Methods

### Model Development

**FIGURE 1:**
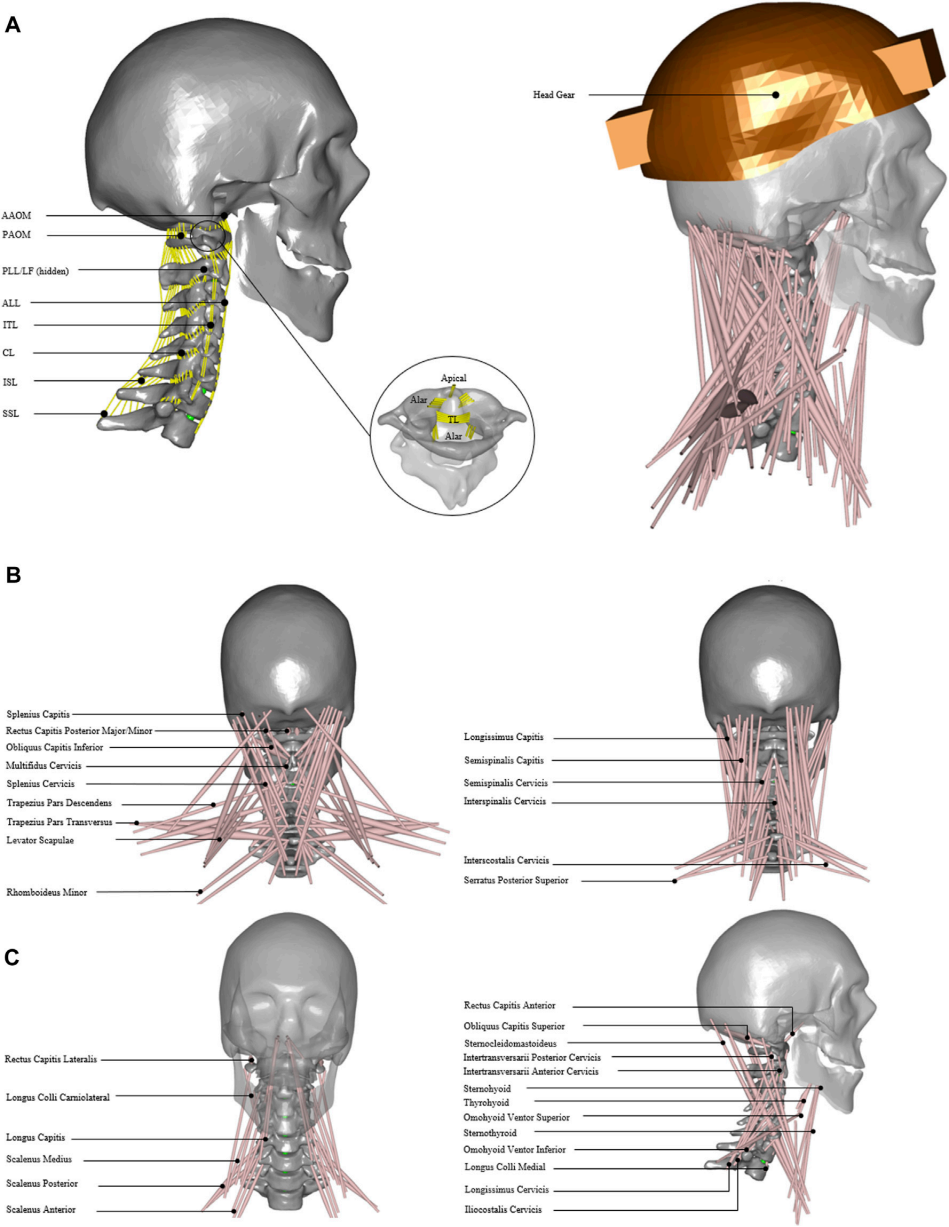
Head and neck model. **(A)** Model with cervical ligament fibers. Apical, alar, TL; transverse ligament, AAOM and PAOM; anterior and posterior atlantooccipital membranes, ALL; anterior longitudinal ligament, PLL; posterior longitudinal ligament, SSL; supraspinous ligament, ISL; interspinous ligament, ITL; intertransverse ligament, CL; capsular ligament, and LF; ligament flavum. **(B)** Model with 34 muscle groups and headgear. **(C)** Detailed front, back, and side view of 34 muscle groups added in the model.

For developing an inverse dynamic musculoskeletal model, the 3D geometry for the head and neck complex was acquired from a previous study (Mesfar and Moglo, 2013) (Figure 1), where they used data from the Visible Human Project (Spitzer et al., 1996; Ackerman, 1998). The male subject’s measured height was 180 cm, which is close to the 50th percentile of Caucasian populations (Cassola et al., 2011).

The center of mass for each vertebral segment was computed for uniform density across the segment volume. The spherical joints were defined between the head–neck complex with three rotational degrees of freedom and were placed at the geometric centroids of the cervical discs. Furthermore, three translational degrees of freedom using force-dependent kinematics (FDK) were added from C2–C3 to C7–T1, allowing soft constraints defined by the stiffness given in the local x, y, and z directions. During inverse analysis, the system resolves the equilibrium equations quasi-statically and computes translations so that the forces in these directions are in equilibrium (Andersen et al., 2011).

The cervical spine ligaments (Figure 1A) such as apical, alar, transverse, anterior longitudinal, posterior longitudinal, supraspinous, interspinous, intertransverse, capsular, ligament flavum, and anterior and posterior atlanto-occipital membranes were added (Table 1). The origin and insertion points for ligaments were taken from a previously developed finite element model of the cervical spine (Mesfar and Moglo, 2013). The nonlinear ligament stiffness properties at a slow strain rate were defined based on previously published experimental data (Shim et al., 2006). The ligament fibers were calibrated for no stress in a neutral posture. The forces did not exceed the maximum computed for 75 percent of the failure strain for the physiological ranges of motion.

**TABLE 1:** Cervical ligament fibers included in the musculoskeletal model.

Type/Level	C0-C1	C0-C2	C1-C2	C2-C3	C3-C4	C4-C5	C5-C6	C6-C7	C7-T1
ALAR		10	6						
APICAL		3							
TL			5						
AAM Ant	13		9						
AAM Pos	13		11						
ALL			5	5	5	5	5	5	5
PLL				5	5	5	5	5	5
SSL			1	1	1	1	1	1	1
ISL			5	5	5	5	5	5	5
ITLL			2	2	2	2	2	2	
ITRR			2	2	2	2	2	2	
CLL	14		14	15	16	16	16	16	15
CLR	14		14	15	16	16	16	16	15
LF				12	12	12	12	12	12
Total	**54**	**13**	**74**	**62**	**64**	**64**	**64**	**64**	**58**

The apical, alar, TL; transverse ligament, AAOM; anterior atlantooccipital membrane, POAM; posterior atlantooccipital membrane, ALL; anterior longitudinal ligament, PLL; posterior longitudinal ligament, SLL; supraspinous ligament, ISL; interspinous ligament, ITLL; intertransverse ligament left, ITRR; intertransverse ligament right, CLL; capsular ligament left, CLR; capsular ligament right and LF; ligament flavum.

The head and neck muscles were added (Figure 1B) based on the previously published dataset of muscle parameters (Borst et al., 2011). In total, 34 muscle groups and 129 muscle fascicles were included (Figure 1C) on each side. These muscle groups were further grouped as anterior/anterolateral, posterior/posterolateral, or lateral muscles (Table 2). The muscle behavior was defined by a simple contractile element with constant specific muscle strength.

**TABLE 2:** Head and neck muscle groups and the number of fascicles (sum of left and right) included in the musculoskeletal model.

Anterior/anterolateral (no)	Posterior/posterolateral (no)	Lateral (no)
1. Rectus capitis anterior (2)	1. Rectus capitis posterior major (2)	1. Rectus capitis lateralis (2)
2. Longus capitis (8)	2. Rectus capitis posterior minor (2)	2. Intertransversarii anterior cervicis (12)
3. Longus colli craniolateral (4)	3. Obliquus capitis inferior (2)	3. Intertransversarii posterior cervicis (12)
4. Longus colli medial (10)	4. Obliquus capitis superior (2)	
5. Sternocleidomastoideus (8)	5. Semispinalis capitis (18)	
6. Scalenus anterior (6)	6. Splenius capitis (14)	
7. Scalenus medius (14)	7. Longissimus capitis (12)	
8. Scalenus posterior (4)	8. Iliocostalis cervicis (6)	
9. Omohyoid venter inferior (2)	9. Intercostalis cervicis (2)	
10. Omohyoid venter superior (2)	10. Interspinalis cervicis (10)	
11. Sternohyoid (4)	11. Splenius cervicis (4)	
12. Thyrohyoid (2)	12. Semispinalis cervicis (20)	
13. Sternothyroid (4)	13. Longissimus cervicis (16)	
	14. Multifidus cervicis (20)	
	15. Levator scapulae (8)	
	16. Rhomboideus minor (4)	
	17. Trapezius Pars descendens (8)	
	18. Trapezius Pars transversus (4)	
	19. Serratus posterior superior (8)	

### Sensitivity Analysis

Three parameters were considered for sensitivity analysis, namely, 1) segmental mass, 2) cervical disc stiffness in compression and shear, and 3) muscle strength. In addition, two external load (EL) conditions (0 and 40 N axial loads on the head) were considered to simulate the head without or with typical headgear, such as in the case of a helicopter pilot wearing a helmet with night vision goggles (NVGs) (Figure 1B). The segmental masses were computed using a scaling function for the 5th, 50th, and 95th percentile implemented in the AnyBody Standing Model (AMMR v. 2.2.3). The axial and shear stiffness were adapted from previous studies (Yoganandan et al., 2001; Dowling-Medley et al., 2020), which were varied as 0.5, 1, and 1.5 of the disc stiffness. For quasi-static inverse analysis, simple muscles were considered with three specific muscle strengths of 30, 60, and 90 N/cm^2^, whereas these values were within the normal range as published in previous literature (Ikai and Fukunaga, 1968; Maganaris et al., 2001). The values set for the three parameters are given in Table 3. In total, 54 simulations were performed for two external load conditions and with parameter settings for 27 models (Supplementary Table S1).

**TABLE 3:** Model parameter values for segmental mass (M), disc stiffness (DS), and muscle strength (MS).

Head and Neck Segment Mass (M) (kg)				Disc Stiffness (DS) (N/mm)								Specific Muscle Strength (MS) (N/cm^2^)		
Segment	5^th^50^th^95^th^			Disc level	Compression			Disc level	Shear			0.5MS1.0MS1.5MS		
	(Estimated from AnyBody Standing Model, AMMR version 2.2.3)				0.5DS1.0DS1.5DS				0.5DS1.0DS1.5DS					
					(Yoganandan et al. 2001)				(Dowling-Medley et al. 2020)					
C0	3.305092	4.434112	5.566028	C2C3	318.75	637.5	956.25	C2-C3 to C7-T1	41	82	123	30	60	90
C1	0.1567685	0.2103207	0.2640103	C3C4	382.65	765.3	1147.95							
C2	0.177671	0.2383635	0.2992116	C4C5	392.3	784.6	1176.9							
C3	0.1707035	0.2290159	0.2874778	C5C6	400.1	800.2	1200.3							
C4	0.163736	0.2196683	0.2757441	C6C7	414.85	829.7	1244.55							
C5	0.163736	0.2196683	0.2757441	C7T1	486.8	973.6	1460.4							
C6	0.1707035	0.2290159	0.2874778											
C7	0.1567685	0.2103207	0.2640103											

### Inverse Analysis

In AnyBody, the muscle and joint forces were calculated by inverse analysis while taking known inertia and external forces into account. To estimate muscle forces in the model, a polynomial-based muscle recruitment criterion was considered that minimizes the muscle stresses with better synergism between neck muscles, which is given in Eq. 1.

$$Minimize\;G=\overset{N}{\underset{i=1}{\sum }}{\left(\frac{F_{i}}{F_{i,max}}\right)}^{\;3},0\leq F_{i}\leq F_{i,max},\;Cf=r.$$ (1)

Here, *G* represents the cost function**,** i represents the muscle number, *N* represents the total number of muscles, $\;F_{i}$ represents the actual muscle force at any instant of the simulation, and $F_{i,max}$ represents the strength of the muscle. The system of equilibrium equations was represented by Cf=r , where f represents a vector of the muscle and joint forces, C represents a matrix of equation coefficients, and r represents a vector of known inertia and external forces.

### Results and Validation/Verification

The intradiscal pressure (IDP) for the cervical discs was estimated as given in Eq. 2.

$$IDP_{\mathrm{mod}el}=\;\frac{F_{c\;\mathrm{mod}el}}{Area_{disc}\;\times CF}.$$ (2)

$F_{c\;model}$ is the estimated axial force from inverse analysis, and $Area_{disc}$ is the cross-sectional area of the cervical discs taken from the literature (Pooni et al., 1986). No studies were available that, in particular, presented the correction factor ( CF ) to estimate IDP in the cervical spine; however, we used the typical CF range (0.55–0.77), with a mean value of 0.66 recommended for the lumbar spine (Dreischarf et al., 2013). The estimated IDP from the model for the 5th, 50th, and 95th percentile population was compared with *in vivo* measurements found in the literature (Kambin et al., 1980; Hattori et al., 1981).

The axial load and translation were taken perpendicular to the surface, and shear force or translation was taken parallel to the surface of the lower segment. The axial and shear loads estimated were compared with the findings in the literature (Barrett et al., 2020). In addition, the estimated 34 group muscle forces and activities were also compared qualitatively with data found in the literature (Assi et al., 2005; Ibrahim, 2015; Van den Abbeele et al., 2018).

## Results

### IDP

**FIGURE 2:**
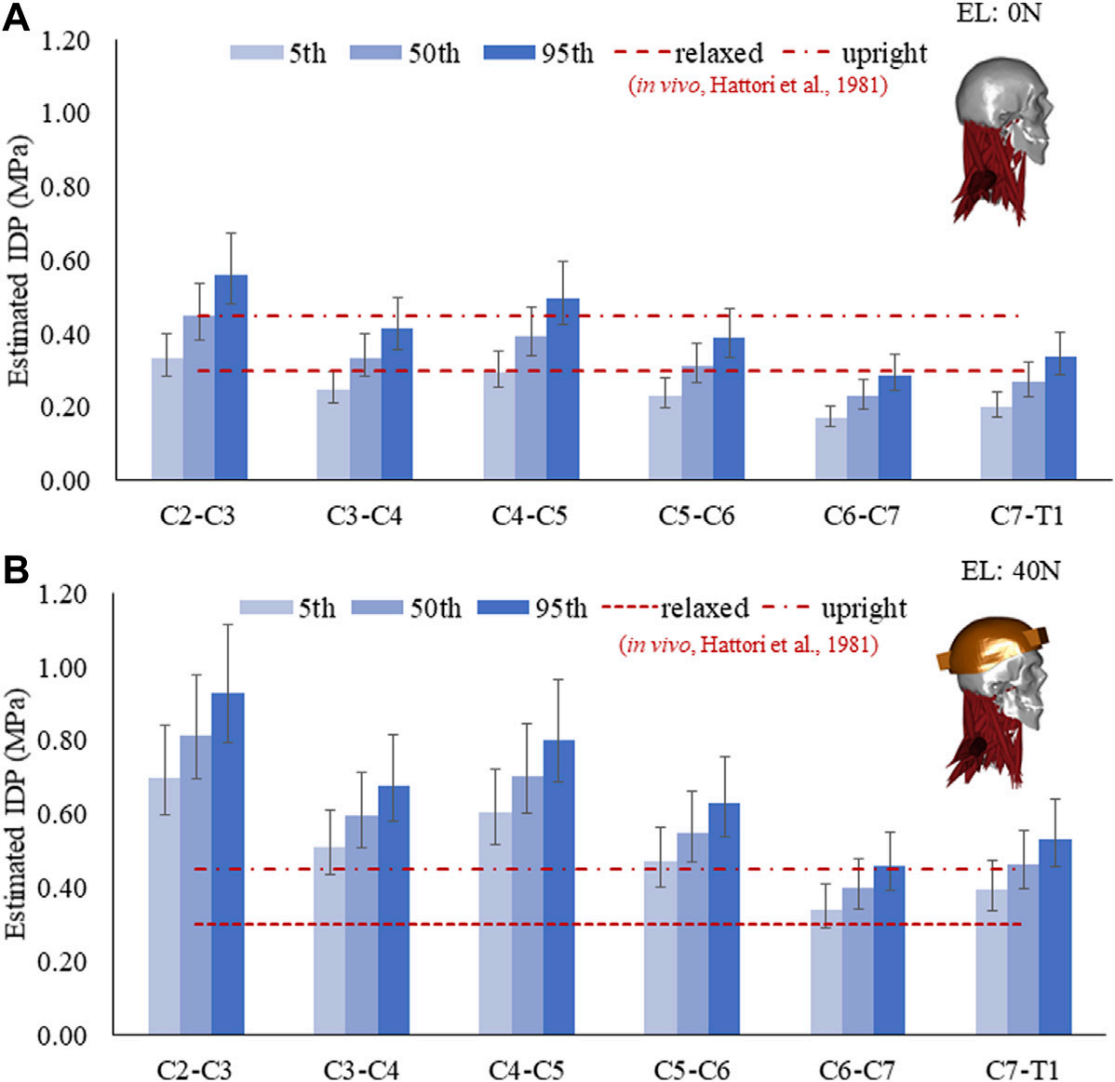
Estimated IDP in a neutral posture for the 5th, 50th, and 95th percentile segmental mass with a mean correction factor of 0.66. Error bars show the range of IDP with a correction factor of 0.55–0.77. **(A)** IDP at 0 N and **(B)** IDP at 40 N external load (EL).

The estimated IDP for C2–C3 to C7–T1 discs and its comparison with *in vivo* measurements are given in Figure 2. With 0 N axial load, the range of average IDP was 0.2–0.56 MPa with a mean correction factor of 0.66 (Figure 2A). These estimations were comparable to *in vivo* measurements (0.3–0.45 MPa). At 40 N axial load, the values increased to 104% (5th percentile mass) and 62% (95th percentile mass), with values in the range of 0.39–0.93 MPa (Figure 2B). Higher values of IDP were estimated in the upper than the lower-level cervical discs. The IDP for the 5th, 50th, and 95th percentile mass showed noticeable differences; however, no significant differences were seen due to disc stiffness and muscle strength. Due to segmental mass, the estimated IDP showed a difference of about 68% at 0 N and 33% at 40 N axial load.

### Disc Loads and Translations

**FIGURE 3:**
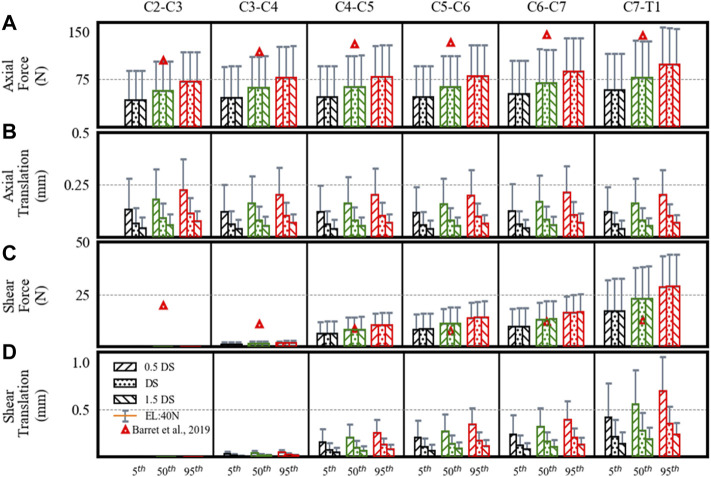
Estimated disc loads and translations in a neutral posture showing differences for the 5th, 50th, and 95th percentile segmental mass, 0.5DS, DS, and 1.5DS disc stiffness (DS). **(A)** Axial force. **(B)** Axial translation. **(C)** Shear force. **(D)** Shear translation. Bars and error bars show disc loads and translations at 0N and 40N external load (EL).

From C2–C3 to C7–T1, the estimated axial loads and their comparison (Barrett et al., 2020) are given in Figure 3A. Barret et al. recorded EMG measurements from eight healthy males wearing a helmet for a helicopter pilot. Furthermore, they predicted the cervical joint loads using an EMG-driven inverse model of a 50th percentile male. In our study, the estimated axial loads for the 50th percentile mass with 40 N external load were comparable to those mentioned in the work by Barret et al.

The axial loads increased from upper to lower levels. At 0 N axial load, the axial loads increased about 68% from the 5th to 95th percentile segmental mass for C2–C3 (43–72 N), C3–C4 (46–78 N), C4–C5 (47–79 N), C5–C6 (47–80 N), C6–C7 (52–88 N), and C7–T1 (58–98 N) segments. For the 40 N axial load, the estimated loads increased by 33% due to segmental mass in C2–C3 (89–119 N), C3–C4 (95–127 N), C4–C5 (96–128 N), C5–C6 (96–128 N), C6–C7 (104–140 N), and C7–T1 (116–156 N) segments. For the 40 N vs. 0 N load condition, axial loads increased by 104–62% for the 5th to 95th percentile mass. No significant differences were found in axial loads due to disc stiffness and muscle strength.

The estimated shear loads are given in Figure 3C. Though the estimated shear loads were small and comparable to those in the work by Barret et al., the shear loads increased from the upper (C2–C3) to the lower level (C7–T1) in our results, which is different from their study (a decrease from C2–C3 to C5–C6, followed by an increase to C7–T1).

Due to segmental mass, the estimated shear loads increased up to 67% for C3–C4 (1–2 N), C4–C5 (6–11 N), C5–C6 (8–14 N), C6–C7 (10–17 N), and C7–T1 (17–29 N) segments. The shear forces computed for level C2–C3 were nearly 0 N. Under 40 N axial load, the estimated loads increased by 33% in C2–C3 (0.2–0.4 N), C3–C4 (2–3 N), C4–C5 (12–16 N), C5–C6 (16–21 N), C6–C7 (19–25 N), and C7–T1 (33–44 N) segments. For the 40 N vs. 0 N load condition, the shear loads increased by 91–52% for the 5th to 95th percentile mass. No notable differences were found due to disc stiffness and muscle strength.

The axial translations at different spine levels were almost similar (Figure 3B), whereas shear translations increased from upper to lower levels (Figure 3D). With an increase in segmental mass, the axial translations increased by 68%. With a decrease in disc stiffness from 1.5 to 0.5, axial and shear translations increased by 67% for each percentile mass. From C2–C3 to C7–T1, the range of axial translations was 0.04–0.22 mm and 0.08–0.37 mm at 0 N and 40 N axial loads, whereas shear translations were between 0 and 0.7 mm and 0–1 mm. For 40 N vs. 0 N load condition, axial translations increased by 104–62% and shear translations increased by 91–52% for the 5th to 95th percentile. No differences were found in disc translations due to muscle strength.

### Muscle Force and Activity

**FIGURE 4:**
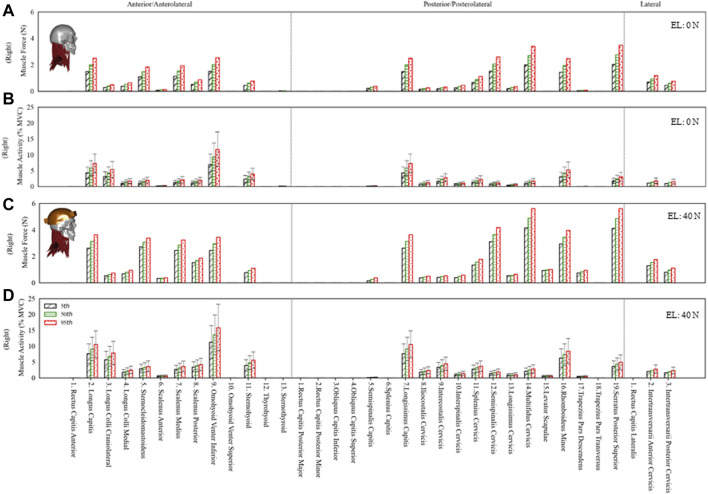
The estimated muscle force and activity of the right side anterior/anterolateral, posterior/posterolateral, and lateral muscle groups for the 5^th^, 50^th^ and 95^th^ percentile segmental mass at 0N and 40N external load (EL). Error bars show variation in muscle activity due to differences in muscle strength from 30 to 90 N/cm^2^. **(A)** Muscle force at 0N EL. **(B)** Muscle activity at 0N EL. **(C)** Muscle force at 40N EL. **(D)** Muscle activity at 40N EL.

**FIGURE 5:**
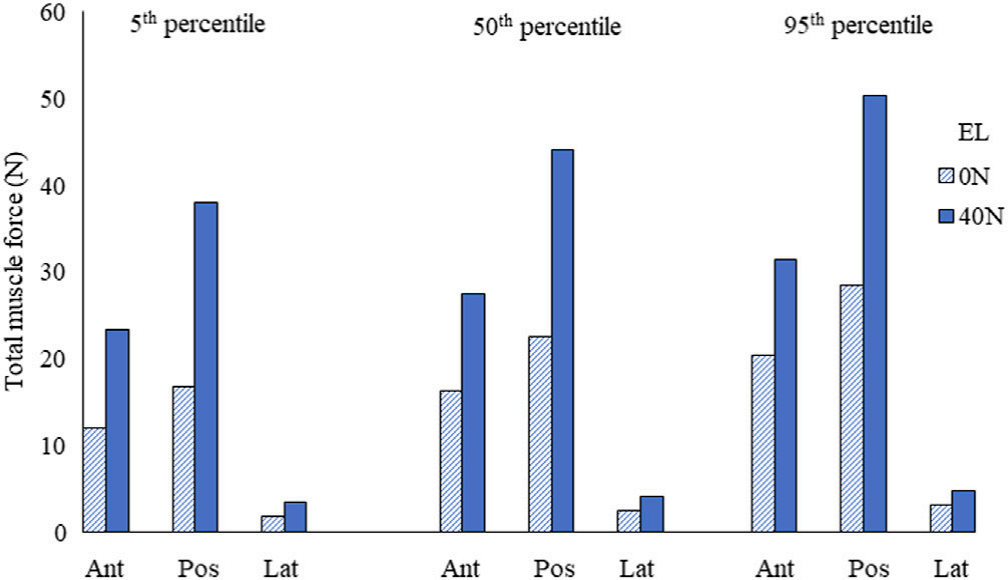
Total muscle force in anterior/anterolateral (Ant), posterior/posterolateral (Pos), and lateral (Lat) muscle groups for the 5th, 50th, and 95th percentile segmental mass at 0 N and 40 N external load (EL).

The estimated muscle force in individual muscle groups for the right side (Figures 4A,C) and the total muscle force in anterior, posterior, and lateral muscle groups (left and right) are given in Figure 5. The maximum muscle forces reached in any individual muscle group were from 3 (0 N axial load) to 5.9 N (40 N axial load). These estimated muscle forces were within the range (approx. 3.2–6 N) found in other studies (Assi et al., 2005; Van den Abbeele et al., 2018).

The total muscle force in posterior muscles was greater than that in anterior muscle groups, whereas the total muscle force in lateral muscles was the least. The muscle forces increased by 69% due to segmental mass under 0 N axial load and 34% under 40 N axial load. Due to 40 N vs. 0 N axial load, the estimated difference in muscle forces was between 103% (5th percentile) and 60% (95th percentile).

The muscle activity for anterior, posterior, and lateral muscle groups is given in Figure 4B,D under 0 N and 40 N axial loads. The average muscle activities were mostly less than 5% of maximum voluntary contraction (MVC), with few up to 10% MVC for 0 N axial load and about 15% MVC for 40 N axial load. The muscle activities estimated in this study with 0 and 40 N axial load for sternocleidomastoid, erector spinae, and trapezius muscles were comparable to the EMG measurements in the study of Ibrahim (2015), where 16 young subjects were measured in a neutral posture with no load and a loaded condition on the head (3.68 kg). Some of the infrahyoid muscle groups showed higher activity in the range of 5–25% MVC. The maximum difference due to segmental mass was about 69 and 34% for 0 and 40 N axial load, whereas the activity varied by 200% due to specific muscle strength. The differences estimated in muscle activity due to disk stiffness varied largely among individual muscle groups.

## Discussion

Due to interindividual differences, the IDP, cervical disc loads, translations, and neck muscle response varies considerably. Quantifying such differences among the general population is necessary for better treatment of musculoskeletal disorders related to the cervical spine. Understanding such differences may help differentiate between healthy and symptomatic population. Sensitivity analysis was performed to investigate the effect of segmental mass, disc stiffness, and muscle strength variation on the axial and shear disc loads, translations, IDP, and muscle force/activity under 0 N and 40 N axial loads in a neutral posture. *In vivo* studies measuring IDP in the cervical discs are rare. We could find two studies in the literature (Kambin et al., 1980; Hattori et al., 1981). Kambin et al. performed intraoperative measurements on 19 patients before a discectomy was undertaken. The disadvantage of intraoperative measurements was the elimination of muscle tone due to the applied anesthetics and relaxants, making it impossible to determine the intradiscal pressure under realistic muscle stress. Therefore, they analyzed the pressure–volume relationship after intradiscal injection of defined fluid measurements. In 62% of the discs, they found normal results with pressure values between 0.6 and 1.2 MPa after injection of 0.2–0.4 ml of fluid. On the other hand, degenerated intervertebral discs with partial rupture of the annulus fibrosus and destroyed nucleus pulposus developed maximum pressures between 0.1 and 0.4 MPa after injection of 1.5 ml of fluid. In analogy, reduced pressures are considered in advanced degenerative changes already proven for the lumbar intervertebral disc (Nachemson, 1966).

Hattori et al. determined the cervical intradiscal pressure *in vivo* in patients who were awake. In 48 patients undergoing treatment for degenerative cervical spine problems, they performed discographic pressure measurements in 80 cervical discs. They took measures in a neutral position on the sitting patient and during flexion/extension, axial rotation, and lateral inclination. In a relaxed, tucked-back position, the values averaged 0.3 MPa, while in a sitting position, they rose to 0.45 MPa in the neutral position.

In addition, a couple of *in vitro* studies (Cripton, 1999; Pospiech et al., 1999) also provided some insight into the cervical IDP under different loading conditions. Pospiech et al. found IDP similar to that found by Hattori et al. in a neutral position from seven specimens (C3–C4 and C5–C6). Cripton, 1999 found a linear relationship between compression and the IDP from a sample of four specimens, including C2–C3 (2), C3–C4 (1), and C4–C5 (1). They found peak IDP of 2.4–3.5 MPa under 800 N. Based on their findings, Cripton et al. suggested a thumb rule that for every 1000 N of axial load, an IDP of 3.75 MPa is expected in cervical discs, which is much higher than that in the lumbar region (1 MPa for every 1000 N). However, in these *in vitro* studies, the sample size was limited and technical difficulties were reported in acquiring IDP for small cervical discs (Cripton, 1999). Numerous biomechanical parameters can affect the cervical disc loads and IDP. In this study, we estimated the change in IDP due to segmental mass in a neutral posture, which was found in a similar range as reported in experimental studies (Kambin et al., 1980; Hattori et al., 1981). The estimated IDP was higher in upper-level discs than in the lower-level discs, possibly due to the smaller size of the upper cervical discs (Pooni et al., 1986).

The estimated cervical disc loads in compression and shear increased from upper to lower cervical levels as the total mass above each level increased naturally. The axial and shear load increased in a neutral posture due to segmental mass; however, it did not change significantly due to disc stiffness and muscle strength. Shear loads were comparatively much less than axial loads, with the highest values at the lowest level and almost no shear load at the upper level of the spine. Here, segmental masses from 5th to 95th percentiles were considered. One may note that even within a specific percentile of the population, the head mass can vary significantly. However, the variation of head mass within a certain percentile was not considered in this study. Due to the small size of cervical discs, the increase in segmental mass and external axial loading showed a considerable rise in IDP. With 40 N axial loading, which is close to a normal auxiliary weight of a helicopter pilot due to a helmet worn with the NVG, our study showed that the IDP could increase two-fold. Previous studies reported the association of a helmet worn with NVG with neck pain in helicopter pilots (Karakolis et al., 2015). High IDP and cumulative loading for persistent long hours might cause tissue damage and, therefore, neck pain.

The cervical disc translations showed significant differences due to segmental mass and disc stiffness. Quantifying the differences in disc translations of the spinal segment may prove helpful in understanding the initial contact mechanics and loading of the facet joints in the neutral posture. The axial translation almost remained similar for different segment levels, which is per increased axial stiffness of the discs that could have compensated the effect of additional mass at progressively lower spine levels. On the contrary, shear translations increased from upper to lower levels as the shear stiffness considered in the model was similar at all levels.

The muscle forces stabilize the head and neck complex in a neutral posture. Here, the sensitivity analysis showed that the activity is much higher with low muscle strength to maintain the neutral posture. While the strength of neck muscles can vary among the normal population, the neck muscles with lower strength can get fatigued earlier for sustaining a posture for a prolonged time. Studies measuring EMG activity reported significant interindividual variations in sternocleidomastoid, erector spinae, and trapezius muscles (Villanueva et al., 1997; Caneiro et al., 2010; Newell et al., 2013; Callaghan et al., 2014; Ibrahim, 2015; Cheon and Park, 2017; Lee et al., 2017). The muscle activities estimated in this study were within a similar range. The estimated muscle activity in neutral posture showed noticeable differences. The maximum muscle activity calculated under 0 N and 40 N axial load was up to 10 and 15% MVC for most muscle groups, respectively. Some infrahyoid muscles showed higher activity (5–25% MVC), which may be associated with their significant contribution in flexion moment. A previous study by Mortensen et al. (2018) showed substantial contribution of hyoid muscles to stabilize the cervical spine by providing increased flexion moment in their model compared to others without hyoid muscles (Vasavada et al., 1998).

The predicted muscle forces in individual groups were approximately in the range of 3–6 N. These values were comparable with those in other studies found in the literature (Assi et al., 2005; Van den Abbeele et al., 2018). Here, we also presented the sum of forces in anterior, posterior, and lateral muscles. The total force in posterior muscles was comparatively higher than that in anterior muscles. Since the overall center of mass was located about C1 and slightly anterior, more extensor moment would be required to keep the spine stable in a neutral posture. With the addition of 40 N axial load, the total muscle forces increased almost two-fold.

This study investigated the differences in cervical disc loads, disc translations, IDP, and muscle force/activity due to segmental mass, disc stiffness, and muscle strength in the general population. However, the study has its limitations. Apart from the effects of the parameters shown in this study, interactions among the parameters may exist. For example, in our preliminary analysis, we noticed negligible or no interaction among the parameters for the disc loads and total muscle forces in a neutral posture; however, for disc translations, nonlinear interaction may exist between the disc stiffness versus segmental mass and the external load. Estimating a full range of variation was not within the scope of this study. Other biomechanical parameters may affect these estimations. Here, we considered only one set of the geometric musculoskeletal model; therefore, differences due to morphological/geometrical parameters were not considered. For example, cervical spine shape varies among the general population as previous studies showed that one-third of the asymptomatic population has cervical kyphosis rather than commonly perceived cervical lordosis (Le Huec et al., 2019, 2014), whereas some studies showed gender differences in the spine shape (Been et al., 2017). In addition, the nonsymmetric geometric features in vertebra shape or bifid in spinous processes may lead to differences in muscle attachments and muscle moment arms. In this study, facet contact mechanics was not considered as it may be sensitive to the definition of facet joint gap and other geometrical parameters. The current model estimated the joint loads, translations, and muscle force/activity in a neutral posture. In our future work, we aim to validate the model for flexion, extension, lateral bending, and axial rotation and include facet joints for simulating these large motions.

## Conclusion

The cervical disc loads, motion, and muscle force/activity vary significantly in a neutral posture. Quantifying such differences due to various parameters is necessary to better evaluate the cervical spine’s normal or pathological condition.

## Data Availability

The original contributions presented in the study are included in the article/Supplementary Material, further inquiries can be directed to the corresponding author.

## Supplementary Material

Supplementary Table 1 Model parameters settings for segmental mass (M), disc stiffness (DS), muscle strength (MS) and external load (EL) for sensitivity analysis.

## References

[B1] Ackerman M. J. (1998). The Visible Human Project. Proc. IEEE 86, 504–511. 10.1109/5.662875

[B2] Ackland D. C. Merritt J. S. Pandy M. G. (2011). Moment Arms of the Human Neck Muscles in Flexion, Bending and Rotation. J. Biomech. 44, 475–486. 10.1016/j.jbiomech.2010.09.036 21074159

[B3] Andersen M. S. Damsgaard M. Rasmussen J. (2011). “Force-Dependent Kinematics: A New Analysis Method for Non-Conforming Joints,” in 13th Biennial International Symposium on Computer Simulation in Biomechanics, Belgium, 30 Jun 2011, 2 Jul 2011 (Leuven: Int. Symp. Comput. Simul. Biomech.).

[B4] Anderst W. J. Donaldson W. F. Lee J. Y. Kang J. D. (2013). Subject-Specific Inverse Dynamics of the Head and Cervical Spine During *In Vivo* Dynamic Flexion-Extension. J. Biomech. Eng. 135, 1–8. 10.1115/1.4023524 PMC3705787 23699719

[B5] Assi A. Pomero V. Bonneau D. Saintonge R. Skalli W. (2005). Cervical Muscles Forces and Spinal Loads Estimation in Standing Position: Asymptomatic Cases. Comp. Methods Biomech. Biomed. Eng. 8, 9–10. 10.1080/10255840512331388001

[B6] Barrett J. M. McKinnon C. Callaghan J. P. (2020). Cervical Spine Joint Loading with Neck Flexion. Ergonomics 63, 101–108. 10.1080/00140139.2019.1677944 31594480

[B7] Been E. Shefi S. Soudack M. (2017). Cervical Lordosis: the Effect of Age and Gender. Spine J. 17, 880–888. 10.1016/j.spinee.2017.02.007 28254673

[B8] Borst J. Forbes P. A. Happee R. Veeger D. (2011). Muscle Parameters for Musculoskeletal Modelling of the Human Neck. Clin. Biomech. 26, 343–351. 10.1016/j.clinbiomech.2010.11.019 21247677

[B9] Bredbenner T. L. Eliason T. D. Francis W. L. McFarland J. M. Merkle A. C. Nicolella D. P. (2014). Development and Validation of a Statistical Shape Modeling-Based Finite Element Model of the Cervical Spine Under Low-Level Multiple Direction Loading Conditions. Front. Bioeng. Biotechnol. 2, 1–12. 10.3389/fbioe.2014.00058 25506051 PMC4245926

[B10] Callaghan J. P. Laing A. C. Dickerson C. R. (2014). The Influence of Neck Posture and Helmet Configuration on Neck Muscle Demands. Technical Report. Available at: https://apps.dtic.mil/sti/citations/AD1000883 (Accessed March 1, 2021).

[B11] Caneiro J. P. O'Sullivan P. Burnett A. Barach A. O'Neil D. Tveit O. (2010). The Influence of Different Sitting Postures on Head/neck Posture and Muscle Activity. Man. Ther. 15, 54–60. 10.1016/j.math.2009.06.002 19643658

[B12] Cassola V. F. Milian F. M. Kramer R. De Oliveira Lira C. A. B. Khoury H. J. (2011). Standing Adult Human Phantoms Based on 10th, 50th and 90th Mass and Height Percentiles of Male and Female Caucasian Populations. Phys. Med. Biol. 56, 3749–3772. 10.1088/0031-9155/56/13/002 21628776

[B13] Catenaccio E. Mu W. Kaplan A. Fleysher R. Kim N. Bachrach T. (2017). Characterization of Neck Strength in Healthy Young Adults. PM&R 9, 884–891. 10.1016/j.pmrj.2017.01.005 28167302 PMC5545075

[B14] Charles L. E. Ma C. C. Burchfiel C. M. Dong R. G. (2018). Vibration and Ergonomic Exposures Associated with Musculoskeletal Disorders of the Shoulder and Neck. Saf. Health Work 9, 125–132. 10.1016/j.shaw.2017.10.003 29928524 PMC6005913

[B15] Cheon S. Park S. (2017). Changes in Neck and Upper Trunk Muscle Activities According to the Angle of Movement of the Neck in Subjects with Forward Head Posture. J. Phys. Ther. Sci. 29, 191–193. 10.1589/jpts.29.191 28265137 PMC5332968

[B16] Ching R. P. (2007). Relationship Between Head Mass and Circumference in Human Adults. Technical Brief. Available at: https://smf.org/docs/articles/pdf/chingtechbrief.pdf (Accessed March 1, 2021).

[B17] Cohen S. P. (2015). Epidemiology, Diagnosis, and Treatment of Neck Pain. Mayo Clinic Proc. 90, 284–299. 10.1016/j.mayocp.2014.09.008 25659245

[B18] Cripton P. A. (1999). Load-Sharing in the Human Cervical. Canada: Queen’s University at Kingston.

[B19] Diao H. Xin H. Dong J. He X. Li D. Jin Z. (20171976). Prediction of Cervical Spinal Joint Loading and Secondary Motion Using a Musculoskeletal Multibody Dynamics Model via Force-Dependent Kinematics Approach. Spine (Phila. Pa. 42, E1403–E1409. 10.1097/BRS.0000000000002176 28368985

[B20] Diao H. Xin H. Jin Z. (2018). Prediction of *In Vivo* Lower Cervical Spinal Loading Using Musculoskeletal Multi-Body Dynamics Model During the Head Flexion/Extension, Lateral Bending and Axial Rotation. Proc. Inst. Mech. Eng. H 232, 1071–1082. 10.1177/0954411918799630 30223718

[B21] Dowling-Medley J. J. Doodkorte R. J. Melnyk A. D. Cripton P. A. Oxland T. R. (2020). Shear Stiffness in the Lower Cervical Spine: Effect of Sequential Posterior Element Injury. Proc. Inst. Mech. Eng. H 234, 141–147. 10.1177/0954411919889194 31749399

[B22] Dreischarf M. Rohlmann A. Zhu R. Schmidt H. Zander T. (2013). Is it Possible to Estimate the Compressive Force in the Lumbar Spine From Intradiscal Pressure Measurements? A Finite Element Evaluation. Med. Eng. Phys. 35, 1385–1390. 10.1016/j.medengphy.2013.03.007 23570899

[B23] Hattori S. Oda H. Kawai S. (1981). Cervical Intradiscal Pressure in Movernents and Traction of the Cervical Spine. Z. Orthop. 119, 568–569.

[B24] Ibrahim E. (2015). The Effects of Neck Posture and Head Load on the Cervical Spine and Upper Extremities. Canada: McMaster University. M.Sc Thesis. Avaliable at: http://hdl.handle.net/11375/18088 .

[B25] Ikai M. Fukunaga T. (1968). Calculation of Muscle Strength Per Unit Cross-Sectional Area of Human Muscle by Means of Ultrasonic Measurement. Int. Z. Angew. Physiol. Einschl. Arbeitsphysiol. 26, 26–32. 10.1007/BF00696087 5700894

[B26] Jaumard N. V. Welch W. C. Winkelstein B. A. (2011). Spinal Facet Joint Biomechanics and Mechanotransduction in Normal, Injury and Degenerative Conditions. J. Biomech. Eng. 133, 071010. 10.1115/1.4004493 21823749 PMC3705911

[B27] Kambin P. Abda S. Kurpicki F. (1980). Intradiskal Pressure and Volume Recording. Clin. Orthopaedics Relat. Res. 146, 144–147. 10.1097/00003086-198001000-00019 7371242

[B28] Karakolis T. Farrell P. Fusina G. (2015). Neck Overuse Injury in CH-146 Griffon Helicopter Aircrews. Proced. Manufacturing 3, 4205–4212. 10.1016/j.promfg.2015.07.396

[B29] Kong L. Tian W. Cao P. Wang H. Zhang B. Shen Y. (2017). Predictive Factors Associated with Neck Pain in Patients with Cervical Disc Degeneration. Medicine (Baltimore) 96, e8447. 10.1097/MD.0000000000008447 29069048 PMC5671881

[B30] Lasswell T. L. Cronin D. S. Medley J. B. Rasoulinejad P. (2017). Incorporating Ligament Laxity in a Finite Element Model for the Upper Cervical Spine. Spine J. 17, 1755–1764. 10.1016/j.spinee.2017.06.040 28673824

[B31] Le Huec J. C. Demezon H. Aunoble S. (2014). Sagittal Parameters of Global Cervical Balance Using EOS Imaging: Normative Values from a Prospective Cohort of Asymptomatic Volunteers. Eur. Spine J. 24, 63–71. 10.1007/s00586-014-3632-0 25344642

[B32] Le Huec J. C. Thompson W. Mohsinaly Y. Barrey C. Faundez A. (2019). Sagittal Balance of the Spine. Eur. Spine J. 28, 1889–1905. 10.1007/s00586-019-06083-1 31332569

[B33] Lee S. Lee Y. Chung Y. (2017). Effect of Changes in Head Postures during Use of Laptops on Muscle Activity of the Neck and Trunk. Ptrs 6, 33–38. 10.14474/ptrs.2017.6.1.33

[B34] Linton S. J. (2000). A Review of Psychological Risk Factors in Back and Neck Pain. Spine 25, 1148–1156. 10.1097/00007632-200005010-00017 10788861

[B35] Maganaris C. N. Baltzopoulos V. Ball D. Sargeant A. J. (2001). *In Vivo* Specific Tension of Human Skeletal Muscle. J. Appl. Physiol. 90, 865–872. 10.1152/jappl.2001.90.3.865 11181594

[B36] McCormick J. R. Sama A. J. Schiller N. C. Butler A. J. Donnally C. J. (2020). Cervical Spondylotic Myelopathy: A Guide to Diagnosis and Management. J. Am. Board Fam. Med. 33, 303–313. 10.3122/jabfm.2020.02.190195 32179614

[B37] Mesfar W. Moglo K. (2013). Effect of the Transverse Ligament Rupture on the Biomechanics of the Cervical Spine Under a Compressive Loading. Clin. Biomech. 28, 846–852. 10.1016/j.clinbiomech.2013.07.016 23972374

[B38] Moroney S. P. Schultz A. B. Miller J. A. A. Andersson G. B. J. (1988). Load-Displacement Properties of Lower Cervical Spine Motion Segments. J. Biomech. 21, 769–779. 10.1016/0021-9290(88)90285-0 3053721

[B39] Mortensen J. D. Vasavada A. N. Merryweather A. S. (2018). The Inclusion of Hyoid Muscles Improve Moment Generating Capacity and Dynamic Simulations in Musculoskeletal Models of the Head and Neck. PLoS One 13, e0199912–14. 10.1371/journal.pone.0199912 29953539 PMC6023174

[B40] Mustafy T. El-Rich M. Mesfar W. Moglo K. (2014). Investigation of Impact Loading Rate Effects on the Ligamentous Cervical Spinal Load-Partitioning Using Finite Element Model of Functional Spinal Unit C2-C3. J. Biomech. 47, 2891–2903. 10.1016/j.jbiomech.2014.07.016 25129167

[B41] Nachemson A. (1966). The Load on Lumbar Disks in Different Positions of the Body. Clin. Orthopaedics Relat. Res. 45, 107–122. 10.1097/00003086-196600450-00014 5937361

[B42] Newell R. S. Blouin J.-S. Street J. Cripton P. A. Siegmund G. P. (2013). Neck Posture and Muscle Activity Are Different When Upside Down: A Human Volunteer Study. J. Biomech. 46, 2837–2843. 10.1016/j.jbiomech.2013.08.013 24095057

[B43] Nguyen A. K. D. Simard-Meilleur A. A. Berthiaume C. Godbout R. Mottron L. (2012). Head Circumference in Canadian Male Adults: Development of a Normalized Chart. Int. J. Morphol. 30, 1474–1480. 10.4067/s0717-95022012000400033

[B44] Panjabi M. M. Crisco J. J. Vasavada A. Oda T. Cholewicki J. Nibu K. (2001). Mechanical Properties of the Human Cervical Spine as Shown by Three-Dimensional Load-Displacement Curves. Spine 26, 2692–2700. 10.1097/00007632-200112150-00012 11740357

[B45] Panjabi M. M. Summers D. J. Pelker R. R. Videman T. Friedlaender G. E. Southwick W. O. (1986). Three-Dimensional Load-Displacement Curves Due to Froces on the Cervical Spine. J. Orthop. Res. 4, 152–161. 10.1002/jor.1100040203 3712124

[B46] Patwardhan A. G. Havey R. M. Ghanayem A. J. Diener H. Meade K. P. Dunlap B. (2000). Load-Carrying Capacity of the Human Cervical Spine in Compression Is Increased Under a Follower Load. Spine (Phila. Pa. 1976) 25, 1548–1554. 10.1097/00007632-200006150-00015 10851105

[B47] Pooni J. Hukins D. Harris P. Hilton R. Davies K. (1986). Comparison of the Structure of Human Intervertebral Discs in the Cervical, Thoracic and Lumbar Regions of the Spine. Surg. Radiol. Anat. 8, 175–182. 10.1007/BF02427846 3099408

[B48] Pospiech J. Stolke D. Wilke H. J. Claes L. E. (1999). Intradiscal Pressure Recordings in the Cervical Spine. Neurosurgery 44, 379–384. 10.1097/00006123-199902000-00078 9932892

[B49] Safiri S. Kolahi A.-A. Hoy D. Buchbinder R. Mansournia M. A. Bettampadi D. (2020). Global, Regional, and National Burden of Neck Pain in the General Population, 1990-2017: Systematic Analysis of the Global Burden of Disease Study 2017. BMJ 368, m791. 10.1136/bmj.m791 32217608 PMC7249252

[B50] Sartori M. Farina D. Lloyd D. G. (2014). Hybrid Neuromusculoskeletal Modeling to Best Track Joint Moments Using a Balance Between Muscle Excitations Derived from Electromyograms and Optimization. J. Biomech. 47, 3613–3621. 10.1016/j.jbiomech.2014.10.009 25458151

[B51] Shim V. P. W. Liu J. F. Lee V. S. (2006). A Technique for Dynamic Tensile Testing of Human Cervical Spine Ligaments. Exp. Mech. 46, 77–89. 10.1007/s11340-006-5865-2

[B52] Silvestros P. Preatoni E. Gill H. S. Gheduzzi S. Hernandez B. A. Holsgrove T. P. (2019). Musculoskeletal Modelling of the Human Cervical Spine for the Investigation of Injury Mechanisms During Axial Impacts. PLoS One 14, e0216663–20. 10.1371/journal.pone.0216663 31071162 PMC6508870

[B53] Spitzer V. Ackerman M. J. Scherzinger A. L. Whitlock D. (1996). The Visible Human Male: A Technical Report. J. Am. Med. Inform. Assoc. 3, 118–130. 10.1136/jamia.1996.96236280 8653448 PMC116294

[B54] Suderman B. L. Vasavada A. N. (2017). Neck Muscle Moment Arms Obtained *In-Vivo* From MRI: Effect of Curved and Straight Modeled Paths. Ann. Biomed. Eng. 45, 2009–2024. 10.1007/s10439-017-1830-8 28397021

[B55] Van den Abbeele M. Li F. Pomero V. Bonneau D. Sandoz B. Laporte S. (2018). A Subject-Specific Biomechanical Control Model for the Prediction of Cervical Spine Muscle Forces. Clin. Biomech. 51, 58–66. 10.1016/j.clinbiomech.2017.12.001 29227919

[B56] Vasavada A. N. Danaraj J. Siegmund G. P. (2008). Head and Neck Anthropometry, Vertebral Geometry and Neck Strength in Height-Matched Men and Women. J. Biomech. 41, 114–121. 10.1016/j.jbiomech.2007.07.007 17706225

[B57] Vasavada A. N. Li S. Delp S. L. (1998). Influence of Muscle Morphometry and Moment Arms on the Moment-Generating Capacity of Human Neck Muscles. Spine 23, 412–422. 10.1097/00007632-199802150-00002 9516695

[B58] Villanueva M. B. G. Jonai H. Sotoyama M. Hisanaga N. Takeuchi Y. Saito S. (1997). Sitting Posture and Neck and Shoulder Muscle Activities at Different Screen Height Settings of the Visual Display Terminal. Ind. Health 35, 330–336. 10.2486/indhealth.35.330 9248215

[B59] Wheeldon J. A. Pintar F. A. Knowles S. Yoganandan N. (2006). Experimental Flexion/Extension Data Corridors for Validation of Finite Element Models of the Young, Normal Cervical Spine. J. Biomech. 39, 375–380. 10.1016/j.jbiomech.2004.11.014 16321642

[B60] Winter D. A. (2009). Biomechanics and Motor Control of Human Movement, Biomechanics and Motor Control of Human Movement. Hoboken: John Wiley & Sons.

[B61] Yoganandan N. Knowles S. A. Maiman D. J. Pintar F. A. (2003). Anatomic Study of the Morphology of Human Cervical Facet Joint. Spine 28, 2317–2323. 10.1097/01.BRS.0000085356.89103.A5 14560077

[B62] Yoganandan N. Kumaresan S. Pintar F. A. (2001). Biomechanics of the Cervical Spine Part 2. Cervical Spine Soft Tissue Responses and Biomechanical Modeling. Clin. Biomech. 16, 1–27. 10.1016/S0268-0033(00)00074-7 11114440

