# Supplementary material for: Sensitivity of the Cervical Disc Loads, Translations, Intradiscal Pressure, and Muscle Activity Due to Segmental Mass, Disc Stiffness, and Muscle Strength in an Upright Neutral Posture
Source: Front Bioeng Biotechnol. 2022 Apr 27;10:751291. doi: 10.3389/fbioe.2022.751291 (PMC9092493; doi:10.3389/fbioe.2022.751291)
Supplement: Supplementary file 1 [file Table1.pdf]

Appendix A

Level	M	Level	DS	Level	MS	Level	EL
1	5th	1	0.5DS	1	0.5MS	1	0
2	50th	2	1.0DS	2	1.0MS	2	40
3	95th	3	1.5DS	3	1.5MS		

Simulation No	M	DS	MS	EL
1	1	1	1	1
2	1	2	1	1
3	1	3	1	1
4	1	1	2	1
5	1	2	2	1
6	1	3	2	1
7	1	1	3	1
8	1	2	3	1
9	1	3	3	1
10	2	1	1	1
11	2	2	1	1
12	2	3	1	1
13	2	1	2	1
14	2	2	2	1
15	2	3	2	1
16	2	1	3	1
17	2	2	3	1
18	2	3	3	1
19	3	1	1	1
20	3	2	1	1
21	3	3	1	1
22	3	1	2	1
23	3	2	2	1
24	3	3	2	1
25	3	1	3	1
26	3	2	3	1
27	3	3	3	1
28	1	1	1	2
29	1	2	1	2
30	1	3	1	2
31	1	1	2	2
32	1	2	2	2
33	1	3	2	2
34	1	1	3	2
35	1	2	3	2
36	1	3	3	2
37	2	1	1	2

Simulation No	M	DS	MS	EL
38	2	2	1	2
39	2	3	1	2
40	2	1	2	2
41	2	2	2	2
42	2	3	2	2
43	2	1	3	2
44	2	2	3	2
45	2	3	3	2
46	3	1	1	2
47	3	2	1	2
48	3	3	1	2
49	3	1	2	2
50	3	2	2	2
51	3	3	2	2
52	3	1	3	2
50	3	2	3	2
54	3	3	3	2
